# Identification of a DNA-cytosine methyltransferase that impacts global transcription to promote group B streptococcal vaginal colonization

**DOI:** 10.1128/mbio.02306-23

**Published:** 2023-10-31

**Authors:** Haider S. Manzer, Tonya Brunetti, Kelly S. Doran

**Affiliations:** 1 Department of Immunology and Microbiology, University of Colorado Anschutz Medical Campus, Aurora, Colorado, USA; Baylor College of Medicine, Houston, Texas, USA

**Keywords:** Group B* Streptococcus*, epigenetics, Dcm, vaginal colonization, DNA methylation, mucin

## Abstract

Group B *Streptococcus* (GBS) colonizes the female reproductive tract (FRT) and causes adverse pregnancy outcomes and invasive disease following vertical transmission to the fetus or newborn. Despite this major public health burden, the mechanisms of GBS FRT colonization are understudied. A recent transposon sequencing screen identified GBS factors contributing to vaginal colonization and ascending spread, including a putative DNA-cytosine methyltransferase (Dcm). We constructed a Δ*dcm* deletion strain and confirmed that *dcm* contributes to murine FRT colonization. Investigation of the evolutionary origin of the *dcm* gene reveals that it is widely distributed across GBS and is encoded as part of a prophage genome that displays evidence of horizontal transfer between GBS strains. We further show that Dcm contributes to 5mC methylation and global regulation of genes involved in carbohydrate metabolism, transcription regulation, and known adhesins and metabolic factors involved in GBS colonization. Interestingly, GBS genes that are induced in the presence of the highly glycosylated vaginal mucin MUC5B were significantly downregulated in the ∆*dcm* mutant. Furthermore, the ∆*dcm* mutant exhibited reduced binding to immobilized mucin and was attenuated in its ability to grow on numerous carbon sources including the carbohydrates found on mucins. While the ∆*dcm* mutant displayed enhanced clearance from the FRT in wild-type mice, there was no significant difference in *MUC5B*^−/−^ mice, indicating that Dcm-mediated regulation requires MUC5B to promote GBS colonization. This is the first report to characterize the impact of a DNA methyltransferase on GBS gene regulation and FRT colonization.

**IMPORTANCE:**

Group B *Streptococcus* (GBS) colonizes the female reproductive tract (FRT) in one-third of women, and carriage leads to numerous adverse pregnancy outcomes including the preterm premature rupture of membranes, chorioamnionitis, and stillbirth. The presence of GBS in the FRT during pregnancy is also the largest predisposing factor for the transmission of GBS and invasive neonatal diseases, including pneumonia, sepsis, and meningitis. The factors contributing to GBS colonization are still being elucidated. Here, we show for the first time that GBS transcription is regulated by an orphan DNA cytosine methyltransferase (Dcm). Many GBS factors are regulated by Dcm, especially those involved in carbohydrate transport and metabolism. We show that GBS persistence in the FRT is dependent on the catabolism of sugars found on the vaginal mucin MUC5B. Collectively, this work highlights the regulatory importance of a DNA methyltransferase and identifies both host and bacterial factors required for GBS colonization.

## INTRODUCTION

*Streptococcus agalactiae* (group B *Streptococcus* [GBS]) is an opportunistic pathogen that asymptomatically colonizes and persists within the female reproductive tract (FRT) of up to one-third of healthy women. GBS can be vertically transmitted to approximately 70% of infants born to GBS-positive mothers (1, 2), 1%–2% of which will develop invasive disease, making GBS a leading cause of global neonatal sepsis, pneumonia, and meningitis (2, 3). Although maternal GBS FRT colonization is typically asymptomatic, the presence of GBS in the vagina is a primary risk factor for numerous adverse pregnancy outcomes. For example, GBS contributes to 1% of all global stillbirths (4) and preterm premature rupture of membranes is 3.6 times more likely during the pregnancy of GBS-positive mothers (5). GBS FRT colonization during pregnancy is also highly associated with the inflammation of intrauterine structures known as chorioamnionitis (6–8). Overall, GBS FRT colonization leads to 409,000 annual cases of infant disease, which results in 147,000 stillbirths and infant deaths worldwide (3). As GBS FRT colonization is the primary risk factor for downstream adverse pregnancy outcomes, transmission to the fetus and newborn, and neonatal invasive disease, a greater understanding of the mechanisms that govern GBS colonization and ascending infection to the uterus is crucial for the development of novel treatment strategies.

Previous studies have sought to identify both bacterial and host factors that contribute to GBS colonization and ascending infection. Several GBS surface adhesins have been shown to promote GBS adherence to human vaginal epithelial cells and murine vaginal colonization. These include the serine-rich repeat proteins (Srr-1, Srr-2), the group B streptococcal surface protein C (BspC), pilus island 2b (PI-2b), plasminogen binding surfacepProtein (PbsP), and BvaP, all of which have been shown to contribute to host cell interaction and colonization *in vivo* (9–14). Many of these have been shown to interact with host receptors such as extracellular matrix components, cytokeratins, and in the case of PI-2b, host mucin (9–12, 15, 16). Interestingly, GBS upregulates pilin genes in the PI-2b locus when exposed to major secreted mucin, MUC5B (11). Other important regulatory mechanisms that contribute to GBS colonization include two-component systems (TCS) such as FspSR, a regulator of carbon metabolism (17), and CovRS, which is known to regulate toxin production to dampen the host inflammatory response and promote GBS persistence (18). We have also shown that Cas9 globally regulates GBS gene expression, including the CiaRS TCS and that both CiaR and Cas9 contribute to GBS vaginal colonization (19). *In vivo* RNA-seq also revealed that the TCS SaeRS is induced during GBS murine vaginal colonization that positively regulates both PbsP and BvaP (13, 14, 20). However, regulatory mechanisms other than TCS have been severely understudied for their potential role in promoting GBS colonization.

In order to investigate additional factors that are required for GBS colonization, we recently performed a murine transposon sequencing (Tn-Seq) experiment using the serotype V, sequence-type 1 strain CJB111 (20). We examined Tn mutants that were underrepresented compared to the input library in vaginal swabs taken from mice at 1- and 3-days post-inoculation, as well as from vagina, cervix, and uterus tissues harvested 3 days post-inoculation. This screen identified manganese homeostasis as being essential for GBS colonization, partially due to its role in mitigating metal stress from host calprotectin (20). Further examination of this Tn-seq data set revealed that mutants with Tn insertions in ID870_06215 were underrepresented in the vaginal lumen as well as in all three FRT tissues. ID870_06215 is annotated as a DNA 5-cytosine methyltransferase (MTase), or Dcm. Here, we investigate how this putative Dcm contributes to GBS colonization of the FRT.

## RESULTS

### Dcm contributes to vaginal colonization and ascending infection

**Fig 1:**
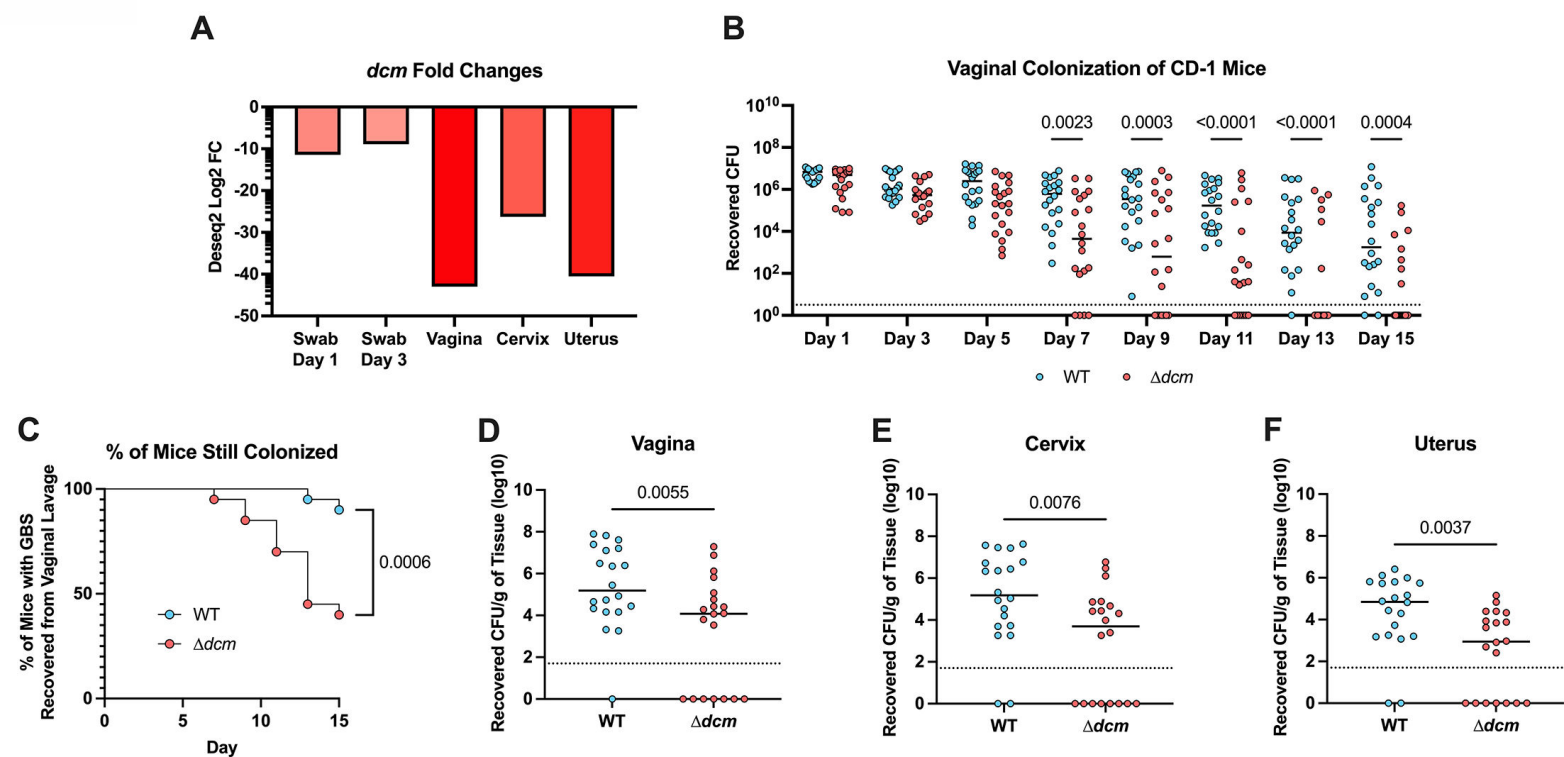
Dcm contributes to GBS vaginal colonization and ascending infection. (**A**) Log2 fold changes of *dcm* from previously published (20) Tn-seq data of GBS colonization taken from vaginal swabs 1 and 3 d.p.i. or FRT tissues taken at 3 d.p.i. as compared to input. (**B–F**) 2 × 10^7^ CFU of WT or ∆*dcm* GBS were inoculated directly into the vaginal tract of CD-1 mice. Recovered CFU counts from vaginal lavage collected every other day (**B**) and the percentage of mice still colonized by each strain (**C**) are shown. On day 15, mice were euthanized, and the vagina (**D**), cervix (**E**), and uterus (**F**) were harvested, homogenized, and plated to enumerate CFU. Panel A shows mean fold changes from three biological replicates. Panels B–F are pooled data from two independent experiments. Each dot represents an individual mouse, and horizontal lines indicate the median. Statistical analysis: panel B: two-Way ANOVA. Panel C: Mantel-Cox log-rank test. Panels D–F: unpaired *t*-test.

Previously published Tn-Seq data from our lab revealed transposon mutants with insertions in ID870_06215 were significantly underrepresented (20). Upon further examination, we found that ID870_06215 was the most significant hit from the vaginal tissue with a 40-fold decrease in recovery, as well as being highly significantly underrepresented at days 1 and 3 in the vaginal lumen and within cervical and uterine tissues harvested 3 days post-infection (d.p.i) (Fig. 1A). The ID870_06215 gene is a predicted orphan Dcm, and these data strongly suggest a role for promoting GBS fitness in the FRT. To investigate this, we generated a ∆*dcm* mutant strain with an in-frame deletion of *dcm* by homologous recombination-driven replacement of the full gene with a spectinomycin cassette. We then used our murine model of vaginal colonization to measure the ability of the ∆*dcm* mutant to persist within the FRT of CD1 mice compared to that of the wild-type (WT) parental strain, CJB111. We observed significantly reduced bacterial burden recovered from the vaginal lumen in mice colonized with the ∆*dcm* mutant as early as 7 d.p.i. (Fig. 1B). Additionally, at the experimental endpoint significantly more mice had cleared the ∆*dcm* mutant (Fig. 1C), and there was a significant reduction in recovery of the ∆*dcm* mutant from vaginal, cervical, and uterine tissues compared to the WT strain (Fig. 1D through F). These results indicate that *dcm* contributes to both colonization and ascension to the uterus. We also repeated this experiment using C57/BL6 mice, where CJB111 exhibits prolonged colonization and observed a similar attenuation of the ∆*dcm* mutant (Fig. S1A through D).

### Contribution of Dcm to 5mC methylation

**Fig 2:**
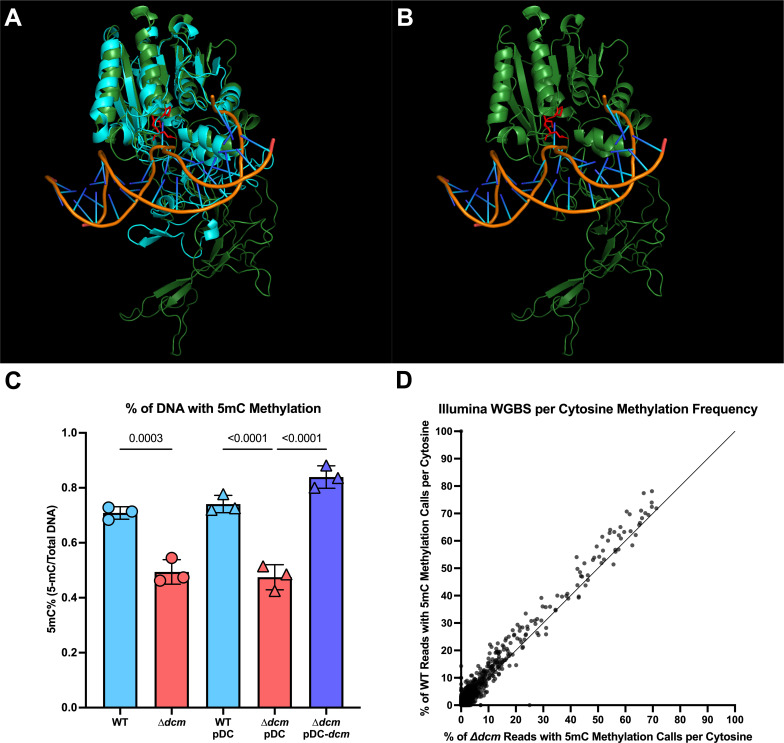
Dcm DNA binding structure and 5mC methylation are conserved. (**A and B**) Alphafold2 (21) was used to generate a predictive model of the GBS Dcm based on the crystal structure of *Haemophilus influenzae* HaeIII Dcm bound to DNA (22). (**A**) Structural alignment of the GBS Dcm predicted structure (green) and the crystal structure of HaeIII (cyan) is displayed by using PyMOL (24). (**B**) CJB111 Dcm with DNA from the HaeIII crystal structure overlayed to display predicted DNA binding. The conserved PCQ motif required for 5mC methylation (25) is shown in red. (**C**) Percentage of total DNA with 5mC methylation from WT, ∆*dcm*, WT + pDC, ∆*dcm* + pDC, and ∆*dcm* +pDC *dcm* is shown as measured by ELISA. Each dot represents the mean of three technical replicates in an independent experiment, bars represent the average of these dots, and the error bars represent the SEM. (**D**) Enzymatic methyl-seq was used to convert non-5mC methylated cytosines to uracils for Illumina whole genome bisulfite sequencing. Bismark was used to identify 5mC methylation. The percentage of reads with a positive methylation call at each cytosine position from WT GBS genomic DNA is graphed against the percentage of reads with a positive methylation call at the same cytosine position in ∆*dcm* GBS genomic DNA.

As this gene was annotated as a DNA 5-cytosine MTase, we next sought to confirm its role in DNA methylation. We began by using AlphaFold2 (21) to predict the Dcm structure, which indicated that the DNA-binding domain and PCQ motif required for methylation were well conserved as compared to the published crystal structure of the *Haemophilus influenzae* Dcm (HaeIII) (Fig. 2A and B) (22). We next compared total genomic methylation content between WT, ∆*dcm*, and the complement strain (∆*dcm* + pDC *dcm*), as well as vector-only (pDC) controls in the WT and ∆*dcm* strains using an enzyme-linked immunosorbent assay (ELISA) specific for 5mC DNA modifications. We observed a statistically significant decrease in 5mC methylation of genomic DNA isolated from the ∆*dcm* mutant, which was restored in the complemented strain (Fig. 2C). We also performed whole genome bisulfite sequencing of WT and ∆*dcm* GBS, using the NEB Enzymatic Methyl-Seq kit for the conversion of methylated cytosines followed by Illumina sequencing and Bismark v0.22.3 for methylation calling. Whole genome bisulfite sequencing allows for the identification of specifically 5mC DNA methylation with single-base resolution (23). Raw reads are available under the Bio Project accession number PRJNA993282. We observed a 33% reduction in the percentage of cytosines that were methylated in the ∆*dcm* mutant as compared to WT GBS (Table 1; Fig. 2D), although this method failed to identify a specific motif associated with methylation. Taken together, these data indicate that Dcm does impact 5mC methylation across the genome, albeit not in a sequence-specific manner.

**TABLE 1:** Whole genome bisulfite sequencing Bismark methylation report

	WT CJB111	∆*dcm* CJB111
Total C’s analyzed	444,686,387	332,854,831
Methylated C’s in CpG context	160,771	94,890
Methylated C’s in CHG context	200,269	109,139
Methylated C’s in CHH context	1,078,858	598,941
Unmethylated C’s in CpG context	55,828,208	41,762,755
Unmethylated C’s in CHG context	68,145,970	51,008,760
Unmethylated C’s in CHH context	319,272,311	239,280,346
% Methylated (CpG context)	0.3	0.2
% Methylated (CHG context)	0.3	0.2
% Methylated (CHH context)	0.3	0.2

To investigate whether Dcm might impact other methylation types, we performed single-molecule real-time (SMRT) sequencing through PacBio. Although PacBio SMRT sequencing is currently unable to detect 5mC methylation, it reliably detects both 6mA and 4mC methylation. We sequenced genomic DNA from the ∆*dcm* strain and compared it to our recently submitted SMRT sequencing of WT CJB111 (BioProject accession number PRJNA977207) to evaluate the differences in methylation patterns (H. S. Manzer and K. S. Doran, submitted for publication). This approach did not reveal any difference in the rates of methylation between WT and ∆*dcm* GBS, nor did it identify any specific motif that was consistently methylated in WT CJB111 and not in the ∆*dcm* mutant, confirming that Dcm does not impact 6mA or 4mC methylation (Fig. S2A and B).

### Dcm regulates GBS transcription

**Fig 3:**
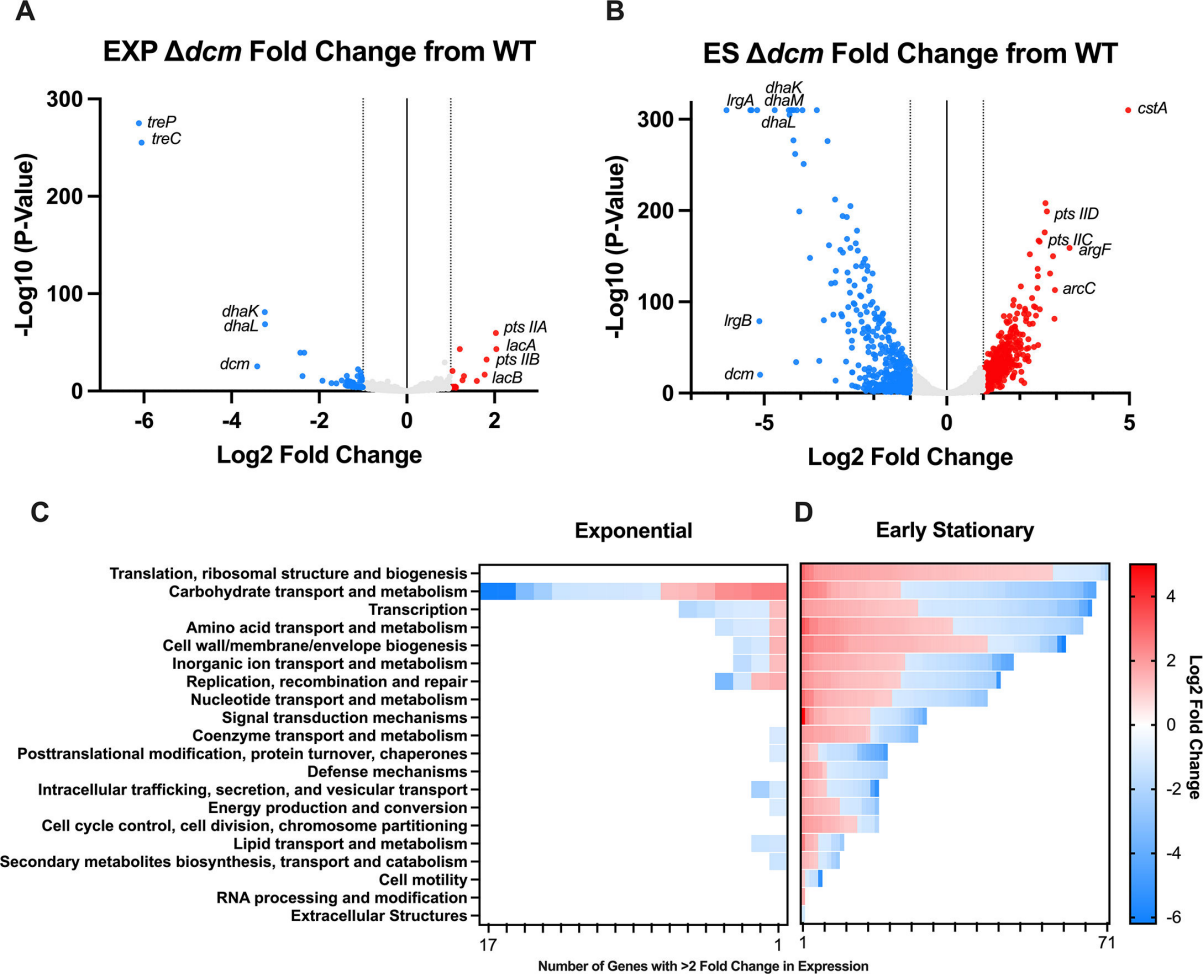
RNA-seq of WT and ∆*dcm* GBS. (**A**) Volcano plot showing genes with altered transcription in the ∆*dcm* mutant during exponential (EXP) growth as compared to WT GBS. (**B**) Volcano plot showing genes with altered transcription in the ∆*dcm* mutant during the early stationary (ES) phase as compared to WT GBS. The number of dysregulated genes at both EXP (**C**) and ES (**D**) growth phases is shown grouped by their COG category and colored by log2 fold change per gene.

Although the *dcm* methylation recognition site is unclear, we hypothesized that *dcm* may still be involved in epigenetic regulation. To identify genes that may be differentially expressed in the ∆*dcm* mutant, we performed transcriptomic analysis using RNA-seq of WT CJB111 and the ∆*dcm* mutant at two growth phases. RNA was taken at an OD 600 of 0.4 to represent mid-exponential growth, as well as at an OD 600 of 1 to represent the early stationary phase. During exponential phase, 14 genes were upregulated, and 46 genes were downregulated in the ∆*dcm* mutant as compared to WT with a fold change of expression > 2 and a *P*-value < 0.05 (Fig. 3A). There were many more changes at the early stationary phase, with 378 genes upregulated and 405 genes downregulated with a fold change of expression > 2 and a *P*-value < 0.05 (Fig. 3B). Analysis of the clusters of orthologous genes (COGs) with altered expression in ∆*dcm* revealed that the COG with the largest number of altered genes in exponential phase was carbohydrate transport and metabolism (Fig. 3C). At the early stationary phase, the COG with the largest number of altered genes was translation and ribosomal structure and biogenesis, followed by COG with genes involved in carbohydrate transport (Fig. 3D). Select genes that were dysregulated are shown in Table 2 and include adhesins, virulence factors, transcription factors, and genes involved in metal homeostasis and carbohydrate metabolism (complete data set shown in Table S1 and raw reads are available under the Bio Project accession number PRJNA993282). Interestingly, some GBS genes already known to be involved in vaginal colonization were downregulated in the ∆*dcm* mutant, including the manganese transport system (ID870_02010–ID870_02020) and the adhesin PbsP (ID870_07365) (14, 20). The genes that were most dysregulated were those involved in sugar metabolism or transport, including *treP/C* (ID870_08455, ID870_08450)*, dhaK/L/M* (ID870_01490–ID870_01480)*, lrgA/B* (ID870_08490, ID870_08485)*, lacA/B* (ID870_00285, ID870_00290)*, argF* (ID870_10075)*, arcC* (ID870_10080)*,* and multiple genes belonging to *pts* sugar transporters. At the early stationary phase, the most significantly upregulated gene in the ∆*dcm* mutant was the carbon starvation protein *cstA* (ID870_04305). qRT PCR was used to validate RNA-seq hits of select genes (Fig. S3A through D). Interestingly, a handful of genes typically considered housekeeping genes such as *rpoB* (ID870_08620)*, rpsL* (ID870_00815)*,* and *gyrA* (ID870_04630) were also significantly dysregulated in the ∆*dcm* mutant during the early stationary phase, but not in the exponential phase. Therefore, 16S was used as the housekeeping gene for these studies as it was unchanged during bacterial growth.

**TABLE 2:** Genes dysregulated in the ∆*dcm* mutant relative to WT

Locus tag	Gene name	Description	EXP fold change	ES fold change
Metabolism				
ID870_08455	*treP*	Trehalose phosphorylase	69.17	2.75
ID870_08450	*treC*	Trehalose-6-phosphate hydrolase	66.48	3.00
ID870_00285	*lacA*	Galactose-6-phosphate subunit A	4.12	5.40
ID870_00290	*lacB*	Galactose-6-phosphate subunit B	3.42	2.82
ID870_01490	*dhaK*	Dihydroxyacetone kinase subunit K	9.46	18.97
ID870_01485	*dhaL*	Dihydroxyacetone kinase subunit L	9.42	19.20
ID870_01480	*dhaM*	Dihydroxyacetone kinase subunit M	5.38	20.00
ID870_04305	*cstA*	Carbon starvation protein A	1.61	31.37
ID870_10075	*argF*	Ornithine carbamoyltransferase	1.16	10.30
ID870_10080	*arcC*	Carbamate kinase	1.17	7.80
ID870_00265		PTS sugar transporter subunit IIA	4.08	2.85
ID870_00270		PTS sugar transporter subunit IIB	3.53	2.27
ID870_00275		PTS galactitol transporter subunit IIC	2.46	1.11
ID870_00200		PTS mannose/fructose/sorbose transporter family subunit IID	1.02	6.68
ID870_07955	*tkt*	Transketolase	1.13	2.96
ID870_08425		Transketolase	1.04	5.04
ID870_08430		Transketolase	1.03	4.82
ID870_01010	*fsa*	Fructose-6-phosphate aldolase	1.07	2.27
ID870_00605		PTS ascorbate transporter subunit IIB	1.47	2.64
ID870_08440		PTS ascorbate transporter subunit IIB	1.16	8.94
ID870_08435		PTS ascorbate transporter subunit IIC	1.41	5.86
Regulators				
ID870_00330		TCS-15 response regulator	1.18	2.33
ID870_03010		TCS-12 HAMP domain-containing histidine kinase	1.27	2.34
ID870_08970	*hrcA*	Heat-inducible transcriptional repressor	2.04	36.43
ID870_00850		MerR family transcriptional regulator	1.27	3.95
ID870_02905		DeoR/GlpR transcriptional regulator	1.08	7.26
ID870_00260		DeoR/GlpR transcriptional regulator	1.71	8.32
ID870_01280		LacI family DNA-binding transcriptional regulator	1.19	2.59
ID870_04400		Spx/MgsR family RNA polymerase-binding regulatory protein	1.12	2.98
ID870_07170		AraC family transcriptional regulator	1.11	2.45
ID870_09330		MurR/RpiR family transcriptional regulator	1.70	2.72
ID870_10525		Helix-turn-helix transcriptional regulator	1.31	2.38
ID870_02005	*mtsR*	Metal-dependent transcriptional regulator	1.09	4.16
ID870_07140	*sczA*	MerR family transcriptional regulator	1.07	3.50
ID870_07405	*copY*	CopY/TcrY family copper transport repressor	1.38	17.43
Metal transport				
ID870_02010	*mtsA*	Metal ABC transporter substrate-binding protein	1.14	18.30
ID870_02015	*mtsB*	Metal ABC transporter ATP-binding protein	1.33	17.77
ID870_02020	*mtsC*	Metal ABC transporter permease	1.72	16.42
ID870_07400	*copA*	Copper-translocating P-type ATPase	1.32	11.26
ID870_07395	*copZ*	Heavy-metal-associated domain-containing protein	1.21	10.28
Adhesins				
ID870_06030		PI-1 major pilin	1.75	5.66
ID870_02595	*pilA*	PI-2a subunit	1.36	4.09
ID870_02600	*pilB*	PI-2a subunit	1.27	2.21
ID870_02615	*pilC*	PI-2a subunit	1.07	2.30
ID870_07365	*pbsP*	YSIRK signal domain/LPXTG anchor domain surface protein	1.67	3.79
Secretion systems				
ID870_04180	*esaA*	Type VII secretion	1.08	4.03
ID870_04185	*essA*	Type VII secretion	1.32	3.47
ID870_04195	*essB*	Type VII secretion	1.13	3.52
ID870_08600		Type II secretion system F family protein	1.57	2.13
Other				
ID870_09380		LysM peptidoglycan-binding domain-containing protein CDS	1.00	6.53
ID870_00925		Class I SAM-dependent MTase	1.16	65.26

**Fig 4:**
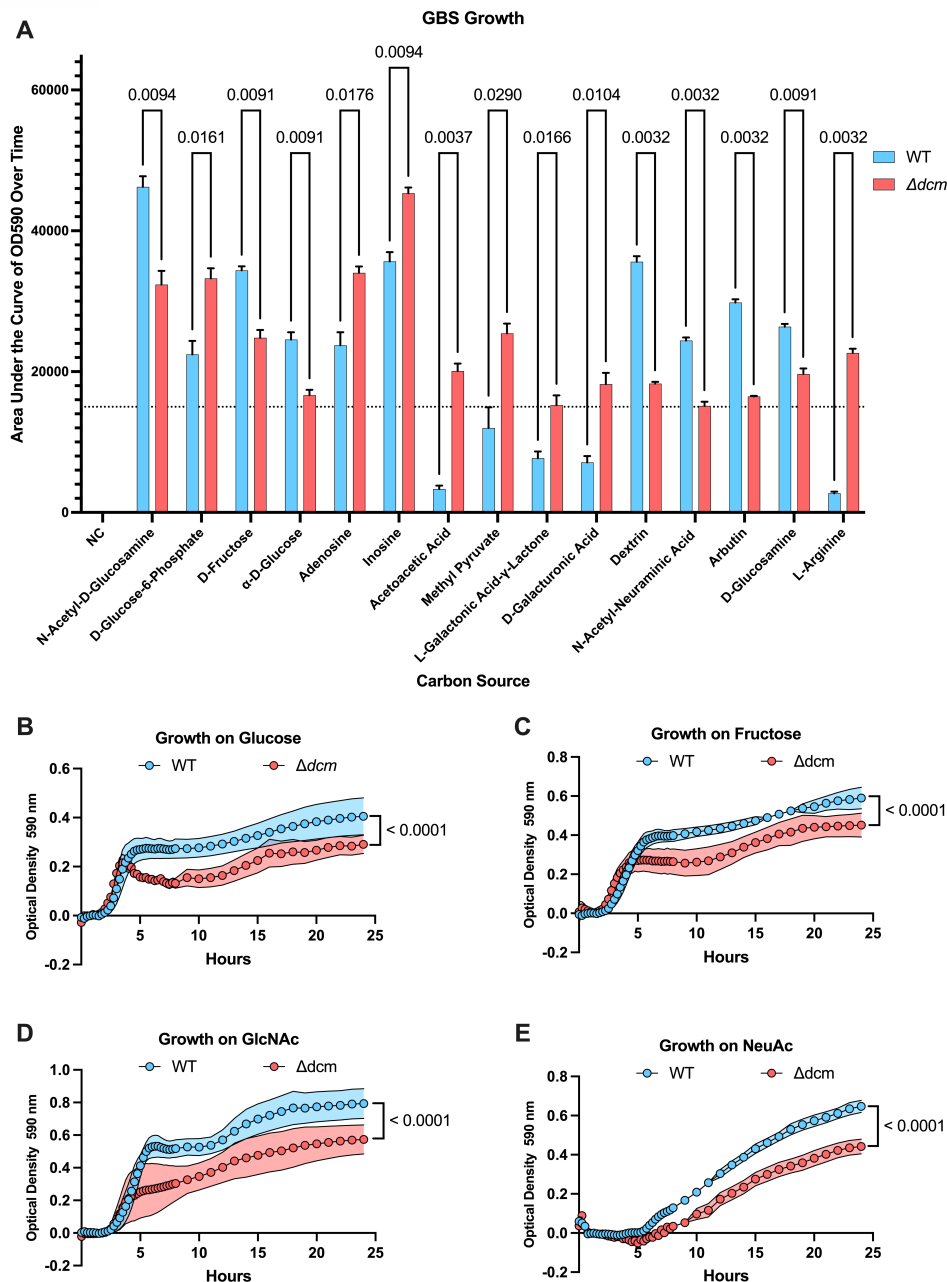
WT versus ∆*dcm* GBS Biolog growth. (**A**) WT and ∆*dcm* GBS were grown in Biolog PM1 and PM2 plates. Area under the curve for each growth curve was calculated. An AUC of 15,000 or greater was arbitrarily selected as a cutoff to filter data to only carbon sources on which either WT or ∆*dcm* GBS displayed significant growth. Only carbon sources on which GBS displayed significant differences in growth between WT and ∆*dcm* are shown. Individual growth curves from Biolog growth assays for glucose (**B**), fructose (**C**), GlcNAc (**D**), and NeuAc (**E**) are shown. All data are pooled from three independent experiments. Statistical analysis: panel A: multiple unpaired *t*-tests. Panels B–E: two-Way ANOVA.

**Fig 5:**
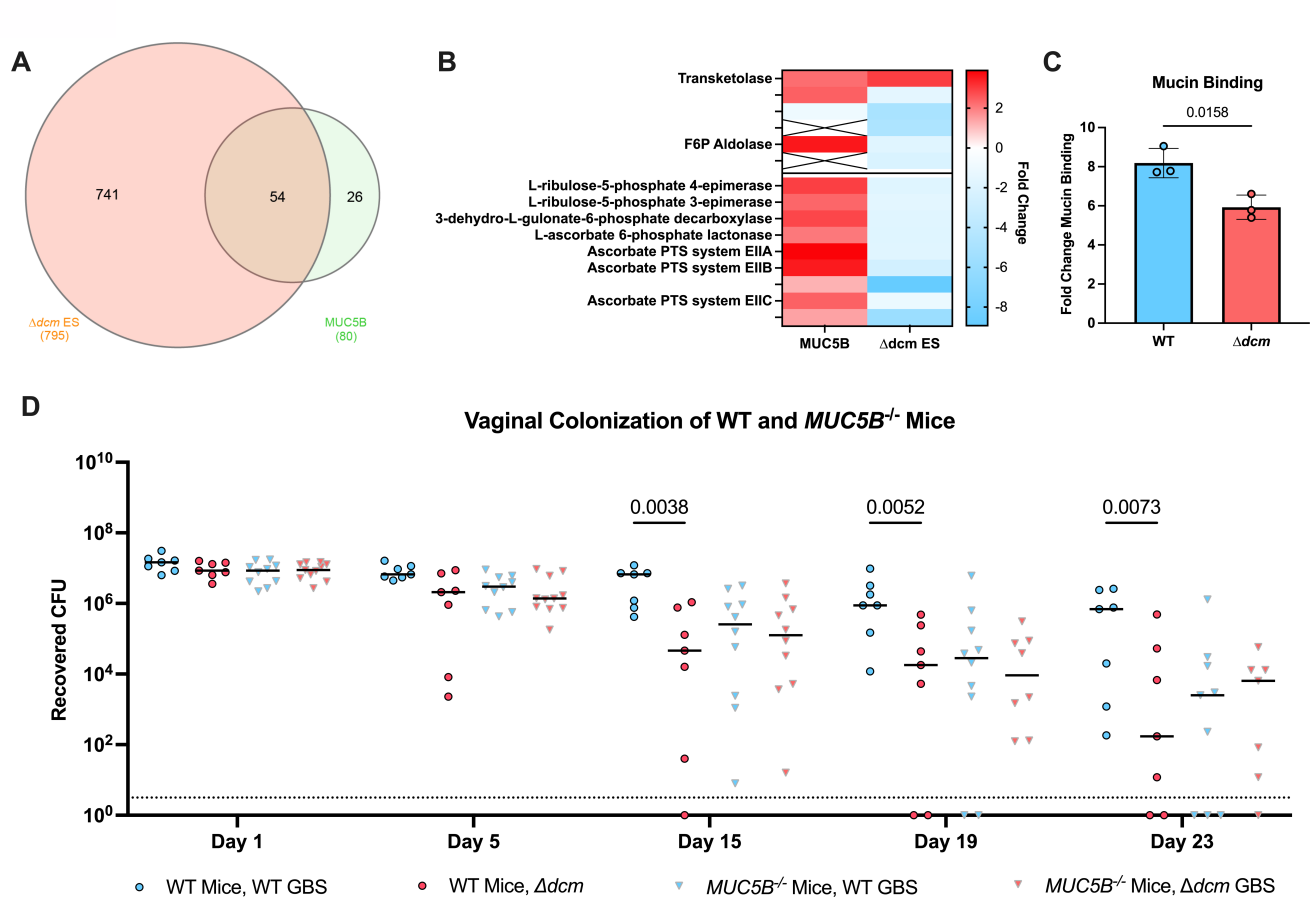
Dcm regulates genes required for MUC5B metabolism. (**A**) Venn diagram showing overlap of genes regulated by Dcm during the early stationary phase and homologs of genes that were previously identified as being upregulated during growth of GBS strain COH1 in the presence of MUC5B (11). (**B**) Comparison of fold changes for genes belonging to the pentose phosphate pathway (top) and ascorbate degradation pathway (bottom) when incubated in the presence of MUC5B as previously published (11) and genes belonging to the same pathways during early stationary growth of the ∆*dcm* mutant. (**C**) Fold change of WT or ∆*dcm* GBS binding to mucin from the bovine submaxillary gland relative to binding to media control wells. Each dot represents the mean of three technical replicates in an independent experiment, bars represent the average of these dots, and the error bars represent the SEM. (**D**) 2 × 10^7^ CFU of WT or ∆*dcm* GBS were inoculated directly into the vaginal tract of WT or *MUC5B^−/−^* C57BL/6 mice. Recovered CFU counts from lavages every other day are shown. Data are pooled from three independent experiments. Each dot represents an individual mouse, with horizontal bars indicating the media. Statistical analysis: panel C: unpaired *t*-test. Panel D: two-Way ANOVA.

As transcription of genes involved with carbohydrate metabolism were the most dramatically altered in the ∆*dcm* mutant, we next sought to determine the functional impact of these changes on the ability of GBS to utilize various carbohydrates. We used the Biolog phenotypic microarray to measure the differences in growth of the ∆*dcm* mutant compared to WT on 190 carbon sources within the Biolog PM1 and PM2A plates (Table S2). Our results for WT GBS closely mimic what was previously observed for this strain, with similar growth observed for each carbon source (17). However, this experiment revealed dramatic differences in the ability of WT and ∆*dcm* GBS to grow on certain carbohydrates as the primary source of carbon (Fig. 4A). For example, the ∆*dcm* mutant displayed significantly decreased growth in glucose, fructose, *N*-acetylglucosamine (GlcNAc), and *N*-acetylneuraminic acid (NeuAc) compared to WT GBS (Fig. 4B through E). Importantly, these are the common carbohydrate moieties found on MUC5B, one of the primary secreted mucins found in the female reproductive tract (11, 26, 27). We previously performed RNA-seq to investigate the impact of MUC5B on GBS gene expression (11). We compared these data sets and found that the majority of GBS genes that were upregulated in the presence of MUC5B are also controlled by Dcm (Fig. 5A). Kyoto Encyclopedia of Genes and Genomes (KEGG) pathway analysis showed that genes belonging to the pentose phosphate pathway and ascorbate degradation were significantly upregulated during MUC5B exposure, while these same genes are downregulated in the ∆*dcm* mutant (Fig. 5B). These data suggest that Dcm may regulate pathways for GBS metabolism of sugars found on vaginal mucin.

### Dcm regulation promotes GBS interaction with mucin

We have shown previously that GBS interacts directly with mucin (11). To functionally investigate the impact of Dcm-dependent gene regulation on this interaction, we first measured the ability of either WT or ∆*dcm* to bind mucin and found that the ∆*dcm* mutant exhibited a significantly reduced ability to bind mucin (Fig. 5C). We observed no difference in adherence to and invasion of human vaginal epithelial cells by WT and the ∆*dcm* mutant (Fig. S4A and B). We next investigated whether MUC5B contributes to the *in vivo* colonization defect of the ∆*dcm* mutant observed previously (Fig. 1B) by vaginally colonizing WT and *MUC5B^−/−^* littermates with WT or ∆*dcm* GBS. We again observed that in WT mice, the ∆*dcm* mutant displayed a significant reduction in the ability to colonize the vaginal lumen (Fig. 5D). Interestingly, bacterial burdens of both WT CJB111 and ∆*dcm* in *MUC5B^−/−^* mice were significantly reduced as compared to WT mice by 19 d.p.i., but there was no significant difference in bacterial burdens or clearance between both strains within *MUC5B^−/−^* mice, indicating that the presence of mucin impacts the *dcm*-mediated phenotype *in vivo*. Collectively, these data suggest that Dcm promotes GBS colonization through the regulation of genes involved in mucin interaction, which supports our previous finding that the presence of mucin is important for optimal GBS colonization (11).

### *Dcm* is a phage-encoded gene distinct from canonical DNA 5mC MTases

**Fig 6:**
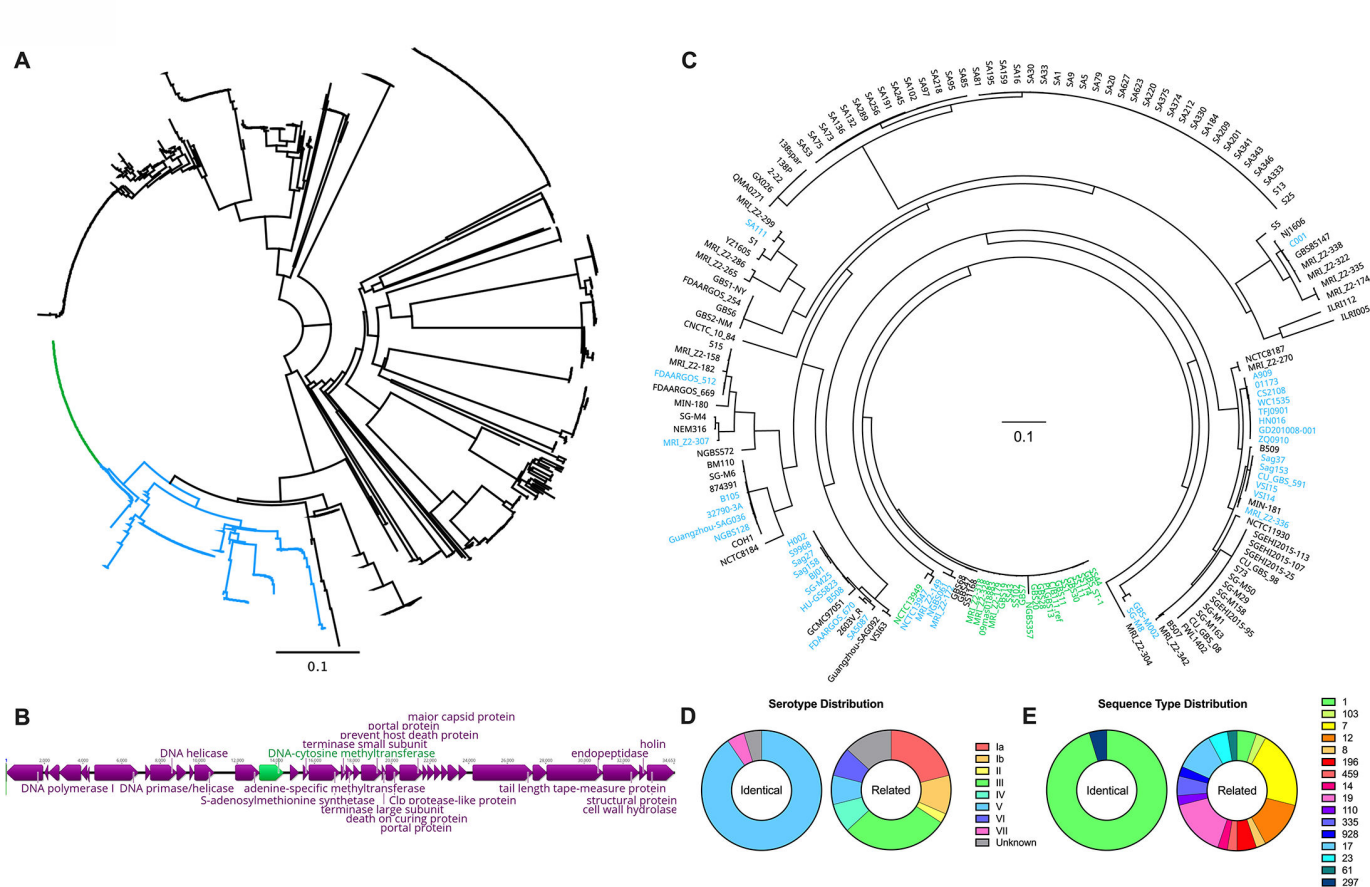
*dcm* is phage-encoded and horizontally transferred across GBS isolates. (**A**) All 917 sequenced GBS genes with a pfam00145 assignment were aligned using MUSCLE and then used to form a neighbor-joining phylogenetic tree. Nodes for genes identical to the CJB111 *dcm* are highlighted in green, and very closely related genes (>85% sequence identity and coverage) are highlighted in blue. (**B**) Representative *dcm*-encoding prophage genome from CJB111 is shown. Unlabeled genes were annotated as hypothetical. (**C**) Harvest Suite and Parsnp were used to generate a core genome alignment and phylogenetic tree from 166 complete GBS genomes currently available on NCBI using CJB111 as the reference genome. Genomes containing a gene that is identical to the CJB111 *dcm* are highlighted in green, and genomes containing a very closely related gene (>85% sequence identity and coverage) are highlighted in blue. The serotype (**D**) and sequence type (**E**) distributions for strains shown in panel C that had either a gene that was identical or very closely related to the CJB111 *dcm* are shown.

To our knowledge, this is the first report characterizing the role of an MTase in GBS colonization. Therefore, we investigated the distribution of *dcm* genes across GBS strains. Dcm is predicted to fall within pfam00145 as a putative C-5 cytosine-specific DNA MTase. As such, we investigated every currently available GBS gene with an associated pfam00145 assignment. Of the 653 GBS genomes currently available from the Joint Genome Institute, 475 (~73%) encode a gene with a pfam00145 assignment. Some of these genomes encode multiple pfam00145 genes, for a total of 917 GBS genes. These genes were aligned and used to generate a phylogenetic tree which revealed the evolutionary clustering of GBS *dcm* genes into three main clades (Fig. 6A). Multiple strains encode multiple distinct pfam00145 genes within one genome. Interestingly, the CJB111 *dcm* is identical to 91 genes and highly similar (>85% sequence identity and coverage) to 233 genes, which collectively account for approximately 35% of all pfam00145 genes in GBS. Further investigation into these conserved genes revealed that they are encoded as part of nearly identical prophage genomes (Fig. 6B; Fig. S5). Additionally, a few clonal strains also displayed a frameshift mutation within *dcm* leading to a premature stop codon after 165 amino acids (Fig. S5). To determine whether the overall distribution of these putative *dcm* genes is clonal or in evolutionarily distant lineages, we constructed a phylogenetic tree based on core genomes of all 166 complete GBS genomes that are currently available through NCBI. While some *dcm-*containing genomes appear to be descended from a common ancestor strain, many of the *dcm-*containing genomes are spread discontinuously across the phylogenetic tree (Fig. 6C). Furthermore, while the serotype and sequence type distributions of strains possessing genes identical to the CJB111 *dcm* are significantly skewed toward serotype V ST-1 strains (*P* < 0.0001 by Fisher’s exact test for both serotype and sequence type distributions), the strains possessing genes that are highly similar but not identical to the CJB111 *dcm* display no trend toward increased prevalence of specific serotypes or sequence types (Fig. 6D and E). Collectively, this evidence strongly suggests that the prophage-encoded *dcm* gene was horizontally transferred between GBS strains.

## DISCUSSION

Here, we show for the first time that an MTase, Dcm, contributes to GBS colonization and ascending infection in the FRT. As an orphan MTase, Dcm is not associated with a cognate restriction enzyme but regulates many GBS factors including those involved in carbohydrate transport and metabolism. While the role of DNA methylation in bacteria has been tightly associated with restriction-modification systems, DNA methylation in eukaryotes is more commonly associated with transcriptional regulation, which has been best studied in humans for its role in cancer (28). There is a growing body of literature indicating that this method of regulation is conserved in bacteria, especially with orphan MTases (29–34). Interestingly, we found that this GBS MTase is encoded within a prophage that appears to have been horizontally transferred between strains. As Dcm regulates a large portion of the GBS transcriptome, including many genes that contribute to human colonization and disease, it is possible that the phage-mediated delivery of this regulatory mechanism may have allowed for GBS adaptation to the human host. The origins of this phage as well as the implications of insertion of a global regulator such as Dcm into the GBS genome warrant further investigation. Furthermore, the observed frameshift mutations indicate that there may be some environments in which Dcm-mediated regulation is deleterious to GBS fitness. Frameshift mutations of MTase genes have been shown to contribute to the adaptation of *Streptococcus pneumoniae* and other pathogens to host niches through phase variation (30, 35–40). Whether frameshift mutations in this Dcm function as a similar reversible modulator of phase variation in GBS is unknown.

DNA methylation has a variety of functional consequences in bacteria, including impacts on biofilm formation, adherence and invasion into various tissues, antibiotic resistance, and virulence (29–34). While the most common form of DNA methylation in eukaryotes is 5mC, it is the least common in bacteria (28, 29, 32, 33, 41, 42). Therefore, the functional impacts of 5mC methylation in bacteria represent an understudied area. Although we showed that Dcm methylation contributes to GBS colonization by regulating the transcriptome, we were unable to identify a DNA recognition motif specifically targeted by Dcm. The 5mC ELISA and whole genome bisulfite sequencing both confirmed Dcm-dependent 5mC DNA methylation and also indirectly confirmed Dcm interaction with DNA, as was predicted by the AlphaFold2 Dcm structural model. Thus, Dcm may also be acting as a transcription factor, and investigations to confirm this hypothesis are ongoing.

Transcription of many genes was dramatically altered in the ∆*dcm* mutant including virulence factors such as secretion systems, adhesins, regulators, and numerous genes involved in carbohydrate transport and metabolism. Multiple genes encoding for transcription factors and TCS regulators were also altered, which may explain the broad dysregulation observed. For example, two genes annotated as DeoR/GlpR transcriptional regulators are significantly downregulated in the ∆*dcm* mutant, with fold changes of −8.3 (ID870_00260) and −7.3 (ID870_02905) at the early stationary phase. DeoR/GlpR transcriptional regulators typically function as global repressors of sugar metabolism; however, there are reports of DeoR/GlpR functioning as an activator in certain species (43–45), which would be more consistent with the general dampening of sugar metabolism observed in the GBS ∆*dcm* mutant. The DeoR/GlpR transcriptional regulators have not been characterized in GBS and represent a potential intermediary between Dcm and transcriptional regulation of sugar metabolism. The DeoR/TetR-like transcription factors associated with the DhaKLM operon of CJB111 are known as DhaS (ID870_01495) and DhaQ (ID870_01500). There was no significant change in the expression of either of these genes, which makes the dramatic changes to the expression of the DhaKLM operon especially interesting. While WT GBS displayed limited growth on dihydroxyacetone (dha) as a sole carbon source, the growth of the *∆dcm* mutant on dha as a sole carbon source was only half that of WT (Table S2). DhaKLM are subunits of the Dha kinase, which are required for the phosphorylation of Dha to Dha-phosphate, which is further utilized during glycolysis. This indicates that Dha metabolism may also be strongly contributing to the growth and colonization defects displayed by the *∆dcm* mutant and should be further investigated. While we focused on carbon sources on which the ∆*dcm* mutant displayed a growth defect, there were multiple carbon sources on which the ∆*dcm* mutant grew significantly better than WT. These include glucose-6-phosphate, adenosine, inosine, acetoacetic acid, galactonic acid-γ-lactone, galacturonic acid, and arginine. Arginine metabolism genes *argF* and *arcC* were the second and third most upregulated genes in the ∆*dcm* mutant at the early stationary phase, so arginine is of particular interest. Arginine metabolism has been linked to bacterial pathogenesis as a mechanism to decrease host antimicrobial nitric oxide production as arginine is a substrate for nitric oxide synthase (46, 47). Arginine metabolism may also benefit GBS via the arginine deaminase pathway by which arginine is converted to ornithine while producing ammonia, CO2, and ATP (47). In addition to the obvious benefit of additional ATP for energy purposes, the conversion of ammonia to NH4^+^ could also serve to increase pH and protect GBS from the acidic conditions of the vaginal tract (48). It is, therefore, possible that Dcm is used to fine-tune gene regulation in response to these additional host defenses as well.

While most of the genes dysregulated in the ∆*dcm* mutant are involved in carbohydrate transport and metabolism, other interesting hits included virulence factors known to promote vaginal colonization. For example, type VII secretion system genes and the PbsP adhesin were downregulated in the ∆*dcm* mutant compared to WT GBS. Interestingly, expression of both Pilus Island 1 (PI-1) and Pilus Island 2a (PI-2a) was significantly upregulated in the ∆*dcm* mutant at the early stationary phase (Table 2; Table S1). While PI-2b has been implicated in promoting GBS adherence and invasion into both epithelial and endothelial cells (49), PI-1 pili do not impact adherence to lung, vaginal, or cervical epithelial cells and instead function to diminish the killing of GBS by macrophages (50). PI-2a, however, has been shown to contribute to GBS adherence and biofilm formation (51, 52). Although the role of PI-2a in interactions with mucin has not been investigated, our observation of a decrease in mucin binding by the ∆*dcm* mutant despite the increased expression of pili warrants further investigation. Interestingly, *pbsP* is potentially the only adhesin-encoding gene that is significantly downregulated in the ∆*dcm* mutant, with a 3.8-fold decrease in expression as compared to WT GBS. *pbsP* has been shown to contribute to GBS vaginal colonization, meningitis, and diabetic wounds through the binding of components of the extracellular matrix such as plasminogen and fibrinogen and adherence to both epithelial and endothelial cells, as is the case with PI-2b (14, 53–57). Potential interactions between *pbsP* and mucin may therefore explain the mucin-binding phenotype observed with the ∆*dcm* mutant and should be investigated further. One of the most significantly changed genes in the ∆*dcm* mutant is ID870_00925, which exhibited a 65-fold decrease in expression at the early stationary phase in the ∆*dcm* mutant compared to WT (Table 2). This gene is annotated as a class I S-adenosyl-L-methionine (SAM)-dependent methyltransferase. While Dcm methylates DNA, class I Rossman fold containing MTases are primarily involved in the synthesis of natural products, which are small molecules that have been widely studied for their potential as antimicrobial therapeutics (58). This type of MTase has not been studied in GBS and warrants investigation.

Within the FRT, MUC1 and MUC4 are the primary cell surface-associated mucins, while MUC5AC and MUC5B are the primary secreted gel-forming mucins (11, 26, 27). Up to 80% of the mass of the secreted gel-forming mucins is composed of various carbohydrates that decorate the mucin protein via *O-*glycosylation (26). The mucus layer provides a physical barrier to hinder bacterial interaction with host cells while also promoting the clearance of pathogens. However, microbes have also evolved many ways of dealing with this host defense. The highly glycosylated nature of mucin led us to investigate the use of the secreted vaginal MUC5B as a carbon source by GBS. Biolog experiments revealed that the ∆*dcm* mutant was less able to grow on multiple sugars found on MUC5B, including NeuAc. Typically, microbes use fucosidases and sialidases to digest common cap glycans fucose or NeuAc, respectively (59, 60). Interestingly, GBS does not encode a functional fucosidase or sialidase and therefore cannot utilize mucin for growth (11). While the pneumococcus encodes the sialidase NanA, the GBS NanA homolog has been evolutionarily rendered inactive and is therefore annotated as “NonA” instead (61). Fucosidase-encoding genes also appear to be absent in GBS. This suggests that GBS alone is not able to degrade mucin for use as a carbon source. However, because we observed MUC5B-dependent phenotypes *in vivo*, we hypothesize that GBS may be able to utilize MUC5B sugars that have already been digested/released by resident vaginal microflora, a phenomenon that commonly occurs in mucosal microbial communities (59). Our lab has previously shown that *Akkermansia muciniphila* increases GBS vaginal persistence (62). *A. muciniphila* readily digests mucins via multiple fucosidases and a sialidase (59, 63, 64), thus, the presence of *A. muciniphila* in the mucosa may release mucin-associated sugars required for growth by other organisms that are unable to utilize them directly, as is the case with GBS. Collectively, this may imply a reliance of GBS on the microbiota to utilize mucin as a carbon source. Additionally, many of the carbohydrates that coat MUC5B are also found on the other vaginal mucins, MUC1, MUC4, and MUC5AC (26, 27). Our Biolog data imply that GBS *dcm*-dependent growth on the carbohydrates present on MUC5B is due to differences in the metabolism of these individual carbohydrates. As such, although we focused on MUC5B here, it is likely that the ∆*dcm* mutant would display similar defects with the metabolism of other mucins found within the FRT as well. In fact, this would likely apply to GBS interactions at all mucosal layers. For example, MUC5B is also expressed in additional host niches where GBS is often found, including the lungs and intestines (26, 65). As GBS also causes neonatal pneumonia and is known to colonize the gastrointestinal tract (1–3), the potential contribution of Dcm to GBS pneumonia and intestinal colonization also warrants further investigation.

Overall, we have shown for the first time a regulatory role for DNA methylation in GBS. We have characterized the impact of this regulation in metabolism as it relates to colonization of the FRT, but the broad nature of the Dcm regulon implies that it likely contributes to GBS virulence in other niches as well. Furthermore, the near-ubiquitous prevalence of DNA MTases across all kingdoms of life indicates that this method of regulation may be an important and understudied mechanism used by all pathogens to colonize and cause human disease.

## MATERIALS AND METHODS

### Bacterial strains and growth conditions

GBS clinical isolate CJB111 (serotype V) (66) and its isogenic ∆*dcm* mutant were used for most experiments and were grown statically in Todd-Hewitt broth (THB) at 37°C. The ∆*dcm* mutant was generated via in-frame allelic replacement with a spectinomycin resistance cassette by homologous recombination as previously described (67). The knockout construct was generated with Gibson assembly of fragments generated using the 5′ flank forward primer, 5′ flank reverse primer overlapping the spectinomycin cassette, spectinomycin forward primer, spectinomycin reverse primer, 3′ flank forward primer overlapping the spectinomycin cassette, and the 3′ flank reverse primer. Nested primers with sequence overlap to pHY304 were used for amplification preceding Gibson assembly into the knockout vector with nested forward primer and nested reverse primer. All primer sequences are provided in Table S3. Primers for amplification of the spectinomycin resistance cassette were used as previously described (68). All other cloning primers were generated in this study. The completed knockout vector was transformed into *E. coli* DH5α for propagation and then transformed into WT CJB111. Integration of the knockout construct and removal of the vector were confirmed via PCR and Sanger sequencing.

The complement forward and complement reverse primers (Table S3) were used to amplify the *dcm* gene from WT CJB111 along with ~250 bp upstream to include the native promoter sequence. Gibson assembly was used to integrate this sequence into the pDCErm expression vector, referred to as “pDC,” which was then transformed into *E. coli* DH5α for propagation before being transformed into the CJB111 ∆*dcm* strain, with PCR and sanger sequencing confirmation. Empty vector controls were generated by transforming pDC directly into WT CJB111 and ∆*dcm* strains, which were confirmed by PCR. pDC was maintained using 250 µg/mL erythromycin (Sigma) for *E. coli* or 5 µg/mL erythromycin (Sigma) for GBS.

### Cell culture and cell-based assays

The well-characterized immortalized human cell line representing vaginal epithelial cells (VK2/E6E7) (69) was obtained from the American Type Culture Collection (ATCC CRL-2616) and was maintained in keratinocyte serum-free media (KSFM) (Gibco) supplemented with 0.5 ng/mL human recombinant epidermal growth factor and 0.05 mg/mL bovine pituitary extract. Cells were grown at 37°C with 5% CO_2_.

Assays to determine the total number of cell surface-adherent bacteria were performed as described previously (67). Briefly, bacteria were grown to the mid-log phase and inoculated onto cell monolayers (1 × 10^5^ CFU, at a multiplicity of infection of 1). Following 30 minutes of incubation, cells were washed 5× with phosphate buffered saline (PBS) to remove non-adherent bacteria. Cells were detached with 0.25% trypsin-EDTA solution and then lysed with 0.025% Triton X-100 by vigorous pipetting. The lysates were then serially diluted and plated on THB agar to enumerate bacterial CFU. Assays to determine the total number of bacteria that had invaded the host cells were similarly performed, except incubation following infection was carried out for 2 hours, after which time the cells were washed once with PBS and fresh media containing 50 µg/mL gentamicin (Sigma-Aldrich) and 2.5 µg/mL penicillin (Sigma-Aldrich). Incubation was continued for two more hours after the addition of antibiotics, and then cells were detached, lysed, and plated to enumerate CFU as described for adherence assays.

### Next-generation sequencing

Genomic DNA from WT and ∆*dcm* was prepared using the Qiagen Gentra Puregene Yeast/Bact. DNA Isolation Kit. For Illumina whole genome bisulfite sequencing, libraries were generated using the NEBNext Enzymatic Methyl-Seq Kit with Covaris shearing according to the manufacturer’s instructions. Sequencing was performed using the NovaSeq 6000 with v1.5 chemistry for 2 × 150 bp reads. Paired-end reads were then quality filtered and trimmed using FastQC, TrimGalore, and Cutadapt. Reads were aligned to the reference genome with deduplication and methylation calling using Bismark v0.22.3.

For PacBio SMRT Sequencing, libraries were prepared using the SMRTbell prep kit 3.0. HiFi sequencing with kinetic data was performed using an SMRT Cell 8M, PacBio Sequel II sequencer, and the SMRTLink v.11 along with primer trimming and demultiplexing. Reads were mapped to reference genome with methylation base calling using SMRTLink v.12.

### 5mC ELISA

Genomic DNA from WT, ∆*dcm*, WT + pDC, ∆*dcm* + pDC, and ∆*dcm* + pDC *dcm* GBS was prepared using the Qiagen Gentra Puregene Yeast/Bact. DNA Isolation Kit, and DNA methylation was quantified using the Epigentek MethylFlash Global DNA Methylation (5-mC) ELISA Easy Kit (Colorimetric) according to the manufacturer’s instructions.

### RNA-sequencing and analysis

Individual colonies of WT and ∆*dcm* GBS were used to seed overnight cultures, which were grown stationary in test tubes at 37°C. Overnight cultures were then used to seed 10 mL of THB at an OD 600 of 0.05 within a flask and grown stationary at 37°C. Triplicate cultures per strain were grown to an OD 600 of 0.4 and the other three cultures were grown to an OD 600 of 1 as measured by a cuvette in a Thermo Scientific Genesys 30 spectrophotometer. RNA was prepared using the Machery-Nagel NucleoSpin RNA isolation kit according to the manufacturer’s instructions, and then sent to SEQCENTER for rRNA depletion sequencing to a depth of at least 12M RNA reads per sample. Reads were mapped to the CJB111 genome (CP063198), and expression values were generated and then used for the calculation of differential expression with DESeq2 using Geneious Prime. qRT-PCR was used to evaluate the transcript abundance of specific genes. All primer sequences are provided in Table S3. Primers for *16S, gyrA, tkt, rpoB,* and *rpsL* were all used as previously described (70).

### Mouse model of vaginal colonization and ascending infection

We utilized our well-established murine model for GBS vaginal colonization (12, 71–74). For various experiments, 7–10-week-old female CD-1 mice from Charles River Labs or C57BL/6 mice from Jackson Labs were used. Seven-to-twelve-week-old female C57BL/6 *Muc5B^−/−^* or wild-type littermate controls were generated and bred for use as described previously (11, 65). The estrus cycles of the mice were synced via intraperitoneal (I.P.) injections of 0.5 mg of β-estradiol in sesame oil. The following day, ~2 × 10^7^ CFU of either WT or ∆*dcm* GBS in 10 µL of PBS was inoculated directly into the vaginal lumen of the mice. Mice were then either swabbed with a sterile ultrafine swab or lavaged with PBS prior to plating on GBS chromagar plates for CFU enumeration. At the experimental endpoint (indicated in figures), mice were euthanized, and their vaginal tract, cervix, and uterus were homogenized and plated on GBS chromagar for tissue CFU enumeration.

### Growth assays

Growth of WT and ∆*dcm* GBS on 190 unique carbon sources was measured using Biolog Phenotypic assays with PM1 and PM2A plates. Growth media for Biolog assays used stock solutions A (488 mg MgCl_2_ and 176 mg CaCl_2_ in 10 mL DI water), B (7.2 mg L-cystine in 30 mL pH 8.5 DI water), and C (2.5 mg lipoamide, 60 mg yeast extract, and 60 µL of tween-80 in 10 mL DI water). From a streptococcal chemically defined medium recipe, 50× amino acid, 100× bases, and 1,000× vitamin stocks were also made (75). Complete Biolog growth media were made fresh before each assay by mixing 100 µL of A, 300 µL of B, 100 µL of C, 240 µL of 50× amino acid stock, 120 µL of 100× bases stock, 12 µL of 1000× vitamin stock, 128 µL of DI water, and 120 µL of Biolog Dye Mix G into 10 mL of Biolog IF0a. WT or ∆*dcm* GBS were grown overnight in THB, washed once with PBS, and then resuspended in Biolog IF0a to an OD 600 of 0.3, and then 880 µL of the bacterial suspension was added to the complete Biolog growth media. A total of 100 µL of this suspension was added per well of Biolog PM1 or PM2A plate. Plates were sealed with DiversifiedBiotech Breathe Easy sealing membranes and then growth was measured using a TECAN plate reader with incubation at 37°C and 5 seconds of agitation before each reading. Data were analyzed by using GraphPad Prism to measure the area under the curve (AUC) for each growth curve. Statistically significant differences in growth between WT and ∆*dcm* were determined by using multiple unpaired Welch’s *t*-tests with the Benjamini, Krieger, and Yekutieli two-stage step-up false discovery approach, with a desired false discovery rate of 5%.

### Mucin binding assay

Mucin binding was measured as previously described (11). Briefly, tissue culture plates were coated with KSFM control or KSFM plus 1% bovine submaxillary gland mucin (Thermo Scientific). Overnight cultures of WT or ∆*dcm* GBS were resuspended in KSFM and used to inoculate coated plates and incubated for 2.5 hours before washing once with PBS and then staining with 0.1% crystal violet for 15 minutes. Crystal violet was then removed, and each well was washed with PBS twice, dried, and then solubilized with 95% ethanol. The plate was agitated for 15 seconds before OD 595 was measured using a TECAN plate reader.

## Data Availability

Raw reads from RNA Seq and whole genome bisulfite sequencing are available under BioProject accession number PRJNA993282.

## SUPPLEMENTAL MATERIAL

Fig. S1 mbio.02306-23-s0001.tif Dcm colonization phenotype is independent of mouse background.

Fig. S2 mbio.02306-23-s0002.tif Dcm does not impact 6mA or 4mC methylation.

Fig. S3 mbio.02306-23-s0003.tif qPCR validation of RNA-Seq.

Fig. S4 mbio.02306-23-s0004.tif Dcm does not impact GBS adherence or invasion to vaginal epithelial cells.

Fig. S5 mbio.02306-23-s0005.tif Alignment of dcm-encoding prophage genomes.

Supplemental Legends mbio.02306-23-s0006.docx Fig. S1-S5 and Table S1-S3 legends.

Table S1 mbio.02306-23-s0007.xlsx RNA-Seq full data set.

Table S2 mbio.02306-23-s0008.xlsx Biolog growth area under the curve full data set.

Table S3 mbio.02306-23-s0009.xlsx Primers used in this study.

ASM does not own the copyrights to Supplemental Material that may be linked to, or accessed through, an article. The authors have granted ASM a non-exclusive, world-wide license to publish the Supplemental Material files. Please contact the corresponding author directly for reuse.
