# Supplementary figures and images for: Identification of a DNA-cytosine methyltransferase that impacts global transcription to promote group B streptococcal vaginal colonization
Source: mBio. 2023 Oct 31;14(6):e02306-23. doi: 10.1128/mbio.02306-23 (PMC10746215; doi:10.1128/mbio.02306-23)

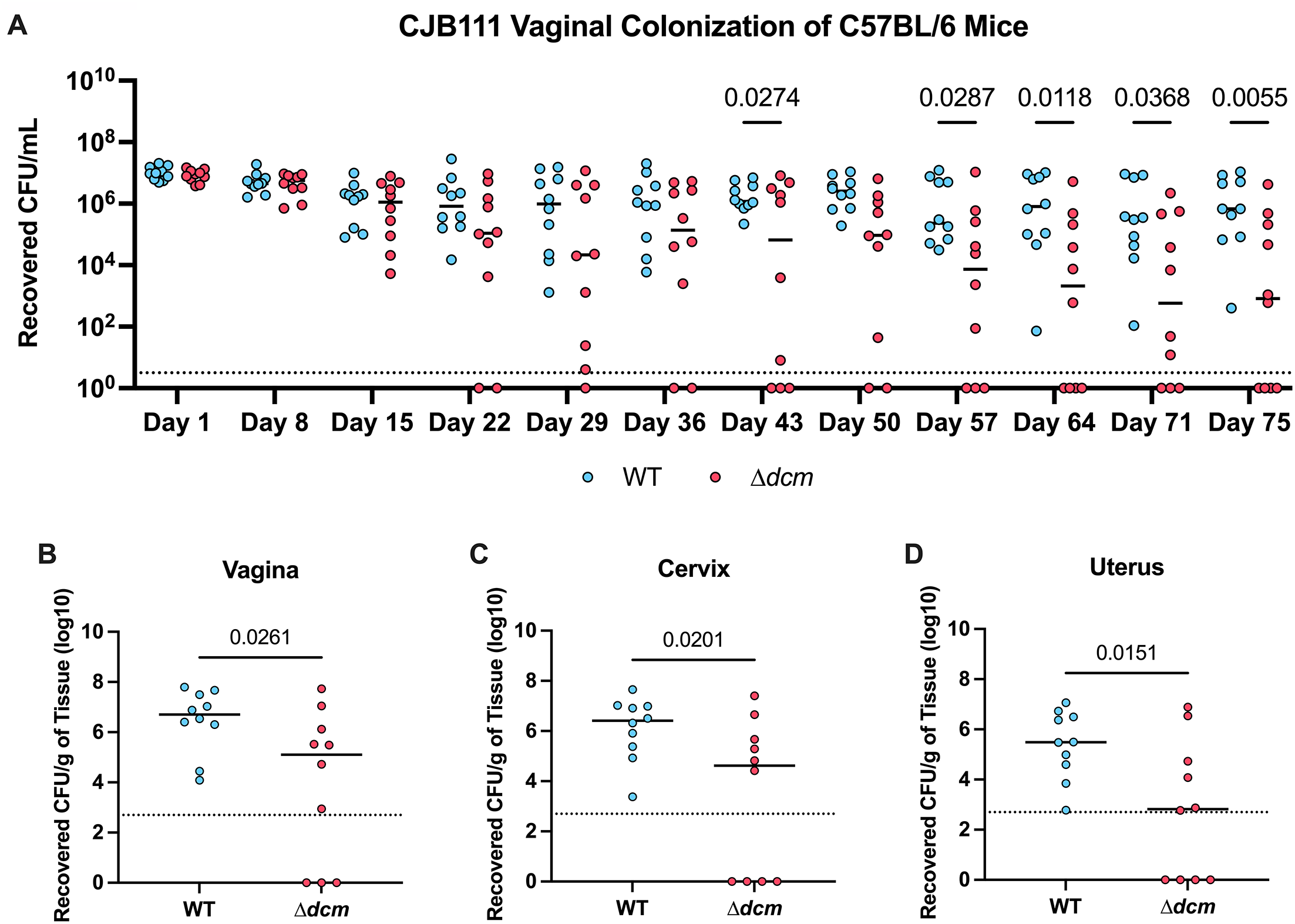

Supplement: Fig. S1 — Dcm colonization phenotype is independent of mouse background. [file mbio.02306-23-s0001.tif]

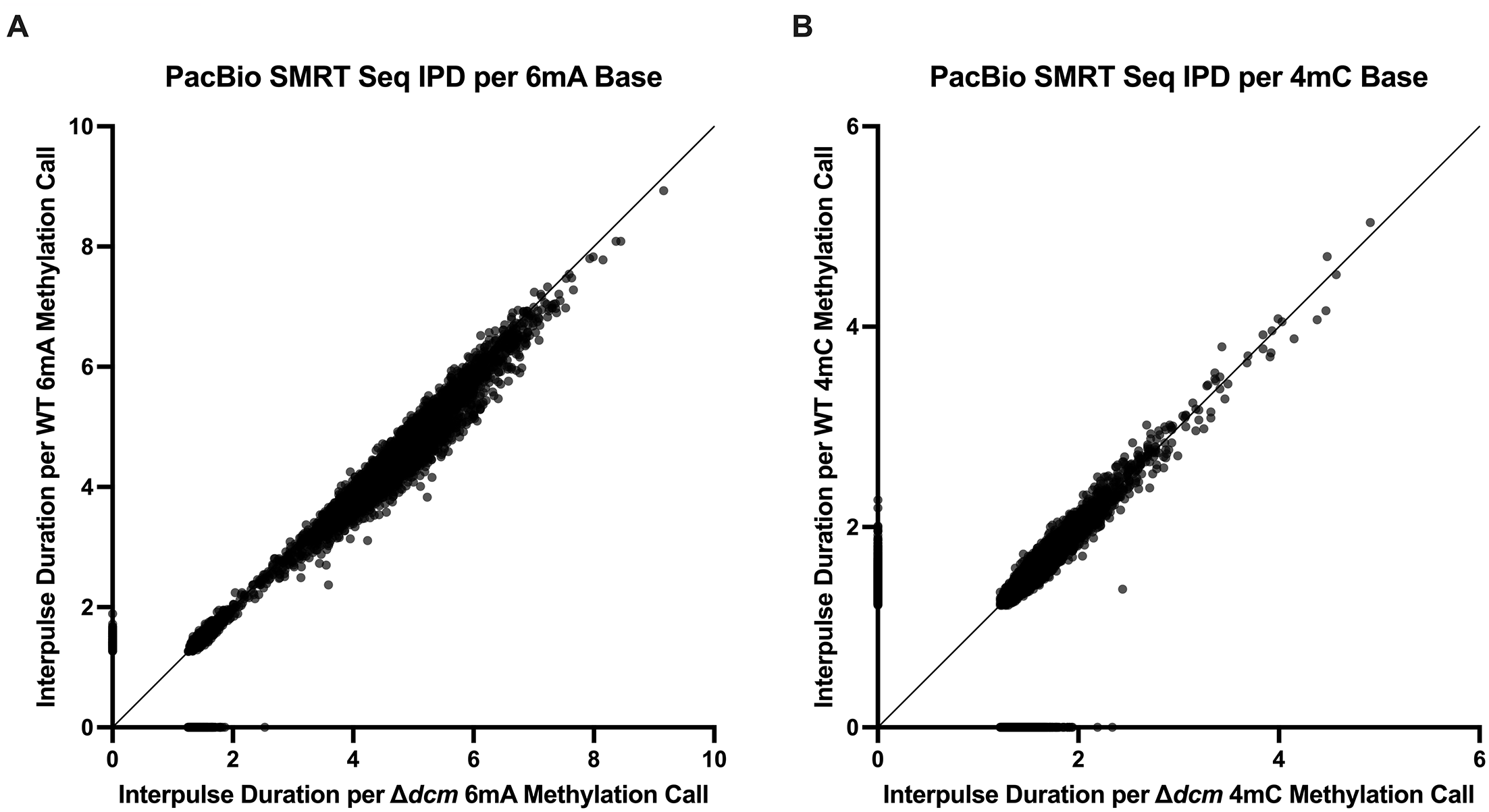

Supplement: Fig. S2 — Dcm does not impact 6mA or 4mC methylation. [file mbio.02306-23-s0002.tif]

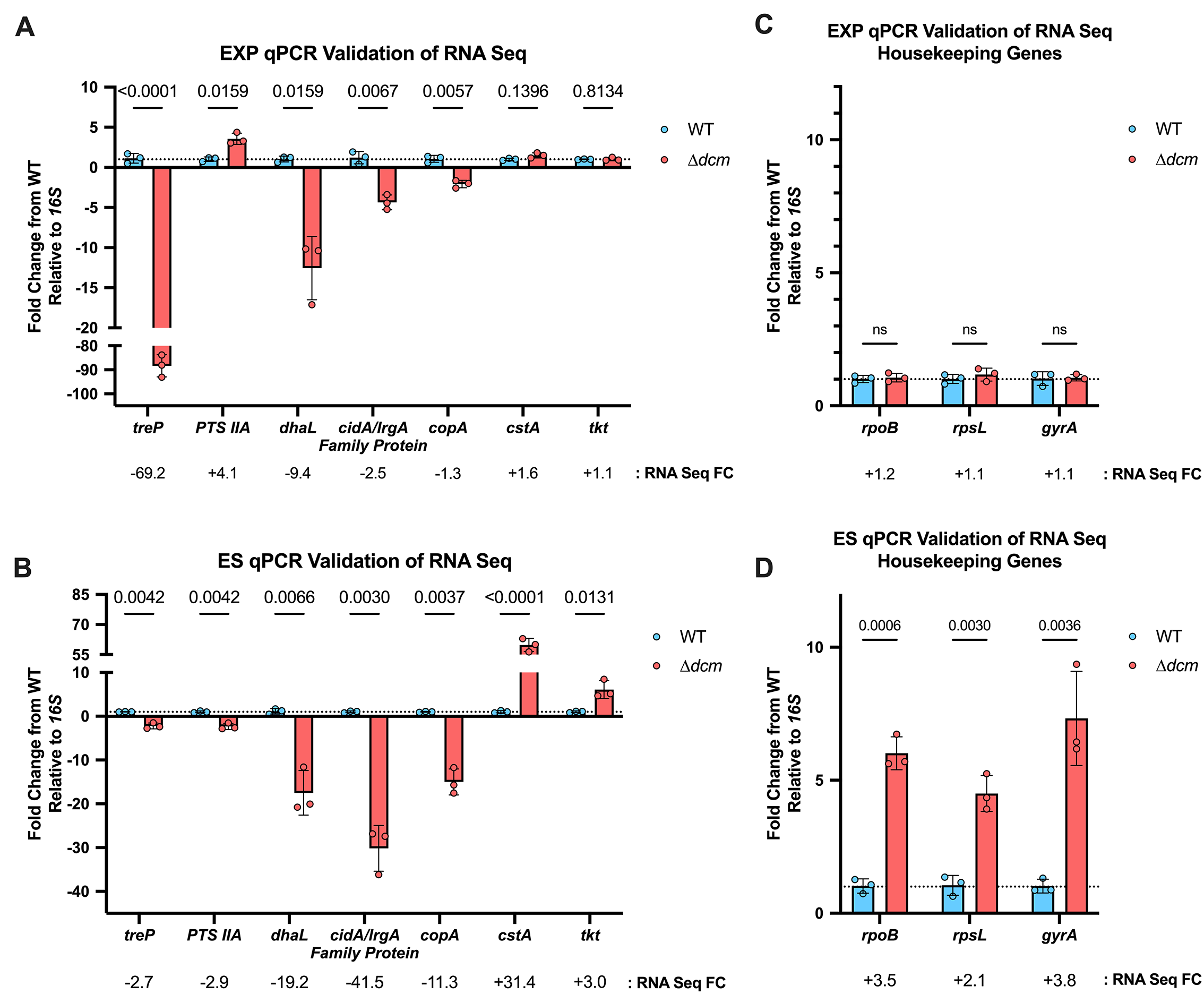

Supplement: Fig. S3 — qPCR validation of RNA-Seq. [file mbio.02306-23-s0003.tif]

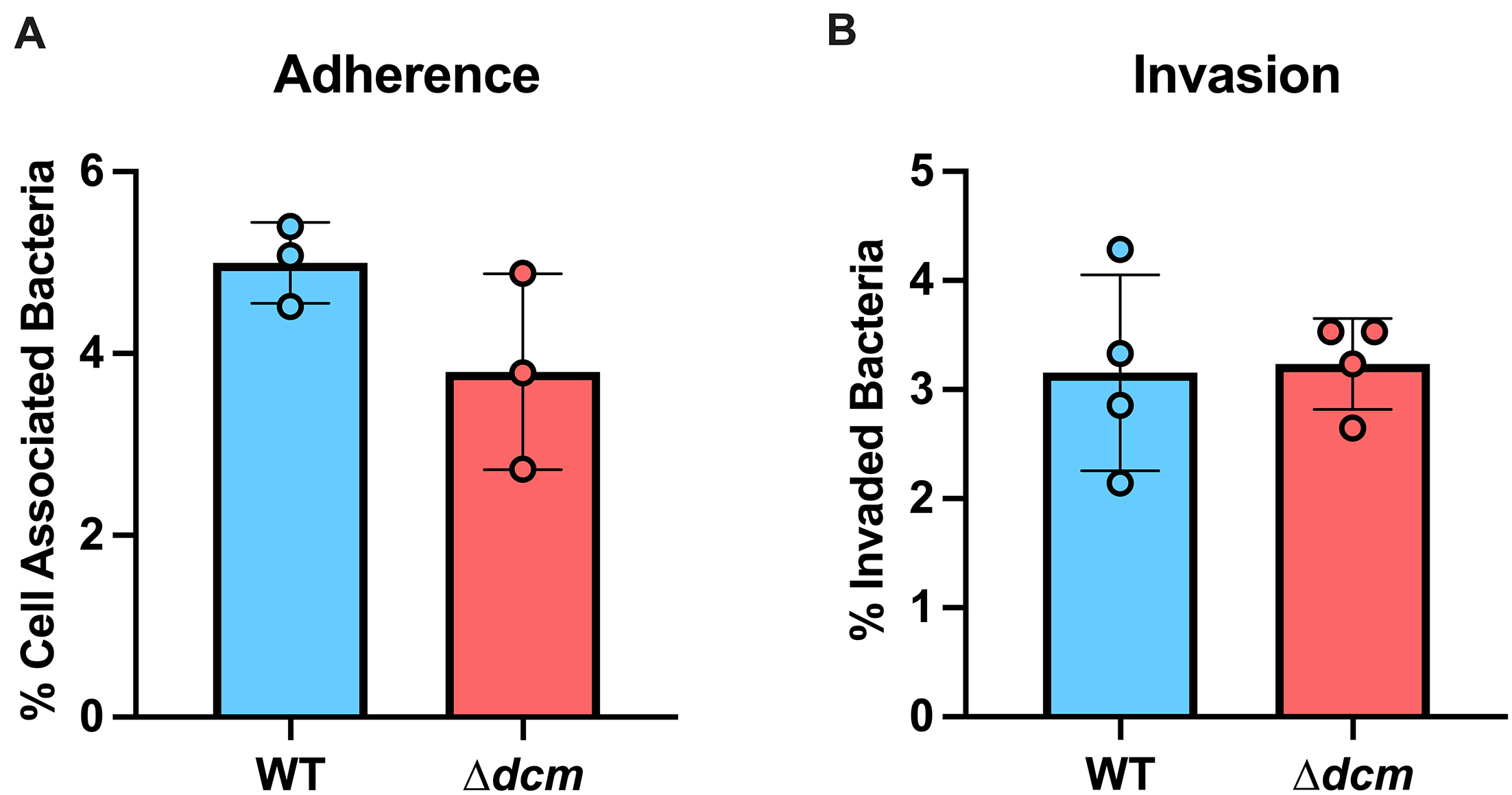

Supplement: Fig. S4 — Dcm does not impact GBS adherence or invasion to vaginal epithelial cells. [file mbio.02306-23-s0004.tif]

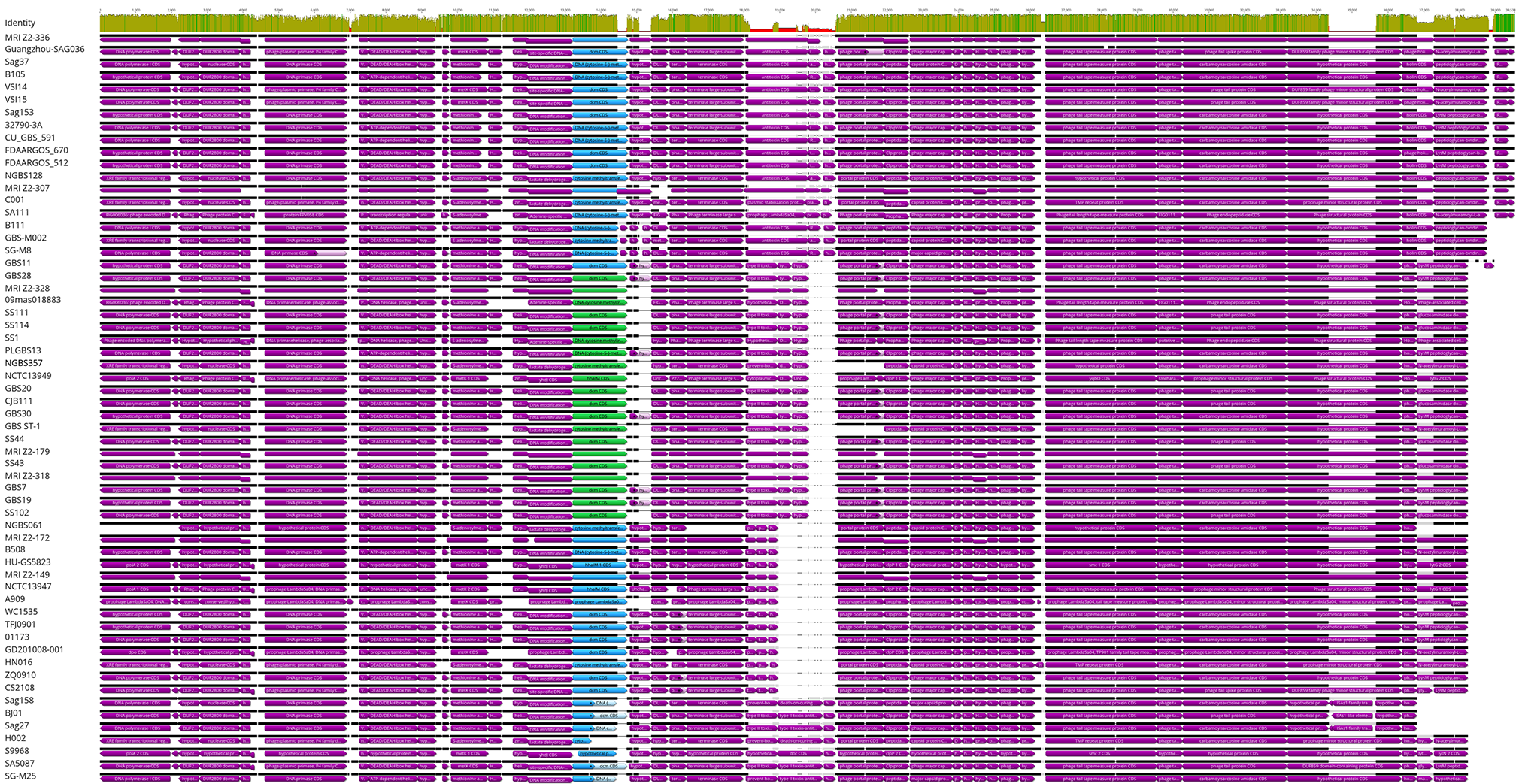

Supplement: Fig. S5 — Alignment of dcm-encoding prophage genomes. [file mbio.02306-23-s0005.tif]
