# Supplementary material for: Identification of a DNA-cytosine methyltransferase that impacts global transcription to promote group B streptococcal vaginal colonization
Source: mBio. 2023 Oct 31;14(6):e02306-23. doi: 10.1128/mbio.02306-23 (PMC10746215; doi:10.1128/mbio.02306-23)
Supplement: Supplemental Legends — Fig. S1-S5 and Table S1-S3 legends. [file mbio.02306-23-s0006.docx]

**Supplementary Figure and Table Legends**

**Figure S1: Dcm Colonization Phenotype is Independent of Mouse Background. (A-D)** 2 × 10^7^ CFU of WT or ∆*dcm* GBS were inoculated directly into the vaginal tract of C57BL/6 mice. **(A)** Recovered CFU counts from lavages every week are shown. At day 75, mice were euthanized, and the vagina **(B)**, cervix **(C)**, and uterus **(D)** were harvested, homogenized, and plated to enumerate CFU. All data is pooled from two independent experiments. Each dot represents an individual mouse and horizontal lines indicate the median. Statistical analysis: A: Two-Way ANOVA. B-D: Unpaired T-test.

**Figure S2: Dcm Does not Impact 6mA or 4mC Methylation.** PacBio SMRT Sequencing was done to identify 6mA **(A)** and 4mC **(B)** methylation. The interpulse duration ratio for each methylation base from WT GBS genomic DNA is graphed against the interpulse duration ratio for the same methylated base.

**Figure S3: qPCR Validation of RNA-Seq.** Expression of select genes at both exponential (EXP) **(A)** and early stationary (ES) **(B)** growth phases was measured using qPCR, with the corresponding RNA-seq fold changes shown at the bottom of each graph. Expression of select genes typically considered housekeeping genes at both EXP **(C)** and ES **(D)** growth phases was measured using qPCR, with the corresponding RNA-seq fold changes shown at the bottom of each graph. Each dot represents the mean of two technical replicates in an independent experiment, bars represent the average of these dots, and the error bars represent the SEM. Statistical analysis: A-D: Multiple unpaired T-tests.

**Figure S4: Dcm Does Not Impact GBS Adherence or Invasion to Vaginal Epithelial Cells. (A)** Adherence of WT and ∆*dcm* GBS to vaginal cells (VK2) was assessed 30 minutes after incubation. **(B)** Invasion of WT and ∆*dcm* GBS into vaginal cells was assessed following 2 hours of incubation, after which antibiotics were added and cells were allowed to incubate for another 2 hours. Each dot represents the mean of four technical replicates in an independent experiment, bars represent the average of these dots, and the error bars represent the SEM. Statistical analysis: A and B: Unpaired T-test.

**Figure S5: Alignment of *dcm-*Encoding Prophage Genomes.** BLAST was used to retrieve prophage genomes containing genes that were identical or very closely related to the CJB111 *dcm,* resulting in 60 hits. These prophage genomes were aligned using Clustal Omega. The genes identical to the CJB111 *dcm* are highlighted in green, while the genes very closely related to it are highlighted in blue. Strains BJ01, Sag27, H002, S9968, SA5087, and SG-M25 all display frameshift mutations.

**Table S1: RNA-Seq Full Dataset.** WT and ∆*dcm* GBS were grown to OD600 0.4 and 1, at which point RNA was harvested and sequenced to measure gene expression. Transcripts were mapped to the CJB111 genome and differential expression was calculated using DESeq2 using Geneious. Fold changes for all genes at both time points are shown. Genes with fold changes greater than 2 with p < 0.05 are can also be viewed in the labeled tabs.

**Table S2: Biolog Full Dataset.** WT and ∆*dcm* GBS were grown in Biolog plates. Growth curves were normalized to the negative control (no carbon source) and the area under the curve (AUC) is shown. Statistically significant differences in growth between WT and ∆*dcm* were determined by using multiple unpaired Welch’s T-tests with the Benjamini, Krieger, and Yekutieli two-stage step-up false discovery approach, with a desired false discovery rate of 5%.

**Table S3: Primer List.** All primers used are listed in the 5’ – 3’ direction.
